# Computational modelling and neural correlates of reinforcement learning following three-week escitalopram: a double-blind, placebo-controlled semi-randomised study

**DOI:** 10.1038/s41398-025-03392-6

**Published:** 2025-05-21

**Authors:** Christelle Langley, Graham K. Murray, Sophia Armand, Franziska Knolle, Rudolf N. Cardinal, Annette Johansen, Peter S. Jensen, Jianfeng Feng, Dea S. Stenbæk, Gitte M. Knudsen, Patrick M. Fisher, Barbara J. Sahakian

**Affiliations:** 1 Department of Psychiatry, University of Cambridge, Cambridge, UK; 2 Behavioural and Clinical Neuroscience Institute, University of Cambridge, Cambridge, UK; 3 Cambridgeshire and Peterborough NHS Trust, Cambridge, UK; 4 Neurobiology Research Unit, Copenhagen University Hospital Rigshospitalet, Copenhagen, Denmark; 5 Department of Psychology, University of Copenhagen, Copenhagen, Denmark; 6 Department of Diagnostic and Interventional Neuroradiology, Technical University of Munich, Munich, Germany; 7 Institute of Science and Technology for Brain-Inspired Intelligence, Fudan University, Shanghai, China; 8 Department of Computer Science, University of Warwick, Coventry, United Kingdom; 9 Department of Clinical Medicine, University of Copenhagen, Copenhagen, Denmark; 10 Department of Drug Design and Pharmacology, University of Copenhagen, Copenhagen, Denmark

**Keywords:** Human behaviour, Neuroscience

## Abstract

Reinforcement learning is a fundamental aspect of adaptive behaviour, since it involves the acquisition and updating of associations between actions and their outcomes based on the rewarding or punishing consequences. Acute experimental manipulations of serotonin have provided compelling evidence for its role in reinforcement learning. However, it remains unknown how more chronic manipulation of serotonin, which holds greater clinical relevance, affects reinforcement learning and the underlying neural mechanisms. Consequently, we aimed to investigate the effect of a three-week administration of the SSRI, escitalopram, on a reinforcement learning paradigm during functional magnetic resonance imaging. The study used a double-blind, placebo-controlled design with 64 healthy volunteers. Participants were semi-randomised, ensuring matched groups for age, sex and intelligence quotient (IQ), to receive either 20 mg of escitalopram (*n* = 32) or placebo (*n* = 32) for at least 21 days. We analysed group differences in reinforcement learning using both analysis of covariance as well as innovative hierarchical Bayesian modelling of the reinforcement learning task. Escitalopram reduced learning from punishment during punishment trials. A key novel finding was that there was decreased activation of the intraparietal sulcus in the escitalopram group when compared to the placebo group during reward trials. The involvement of the intraparietal sulcus suggests that escitalopram affects the encoding of value outcome, which may lead to reduced reinforcement sensitivity, and thereby impacting adaptive learning from feedback. Understanding these mechanisms may help to optimize SSRI treatment to mitigate clinical symptoms and improve quality of life for neuropsychiatric patients, by elucidating serotonin’s effects on affect, cognition, and behaviour.

## Introduction

Serotonin or 5-hydroxytryptamine (5-HT) is a monoamine neurotransmitter known for its multifaceted role in regulating mood, cognition and behaviour [1, 2]. Reinforcement learning is a key component of adaptive behaviour, which is essential in everyday life, therefore, understanding the role of serotonin in this process is of particular importance. Abnormal response to reinforcing feedback has been noted in neuropsychiatric conditions and in particular in major depressive disorder (MDD) [3–5]. Specifically, individuals with MDD often show exaggerated responses to negative feedback, whereas they fail to respond to rewards appropriately [3, 5, 6]. This suggests that there is an impairment in responding to rewarding and punishing feedback. Similarly, individuals with MDD seem to have a negative bias to emotional stimuli, where they respond faster to negative compared to positive emotional stimuli [7, 8]. Studies have shown that serotonin ameliorates these negative biases in depressed patients, and also enhance the positive bias in healthy volunteers [9]. Considering that drugs targeting the serotonin system such as selective serotonin reuptake inhibitors (SSRIs) are the first-line pharmacological treatments for MDD [10], it is of importance to determine whether serotonin plays a role in learning from reward and punishment. Understanding how serotonin modulates reinforcement learning processes has significant implications for elucidating the mechanisms underlying various neuropsychiatric disorders characterized by dysregulated reinforcement learning.

Reinforcement learning is a fundamental aspect of adaptive behaviour. It involves the acquisition and updating of associations between actions and their outcomes based on the rewarding or punishing consequences [11]. The involvement of serotonin in reward learning is evident through the widespread distribution of serotonergic receptors in brain regions implicated in reward processing, including the mesolimbic dopamine system, prefrontal cortex, and amygdala [12]. Moreover, serotonergic projections innervate key nodes of the reward circuitry, exerting intricate regulatory effects on synaptic transmission and neural activity.

Experimental manipulations of serotonin have provided compelling evidence for the involvement of serotonin in reinforcement learning, yet findings vary depending on the specific manipulation employed and the duration of administration. Studies utilizing tryptophan depletion, the precursor to serotonin, have consistently demonstrated impaired performance on reinforcement and reversal learning tasks [13–16]. Similarly, investigations into SSRI administration, both acute and chronic, have revealed significant effects on reinforcement learning processes [17–21].

Remarkably, serotonin may exert differential effects on learning from rewards and punishments. For instance, studies have indicated that tryptophan depletion selectively affects punishment prediction, sparing reward prediction [13]. On the other hand, after boosting serotonin, Michely et al. [19] demonstrated that sub-chronic (7 day) SSRI administration enhanced learning from punishment while reducing learning from reward. However, contrasting findings from animal studies suggest an opposing pattern, where increasing serotonin in rats decreased sensitivity to negative feedback but heightened reward sensitivity [22]. Furthermore, some investigations propose that there may be no inherent asymmetry in learning following serotonin manipulation in healthy humans [20, 21]. For instance, Scholl et al. [21] found that two weeks of SSRI administration improved learning from both rewards and punishments. ROur own recent research [20] has shed further light on the effects of three-week SSRI administration on reinforcement learning using a reversal learning task. Contrary to previous studies, they reported that reinforcement learning rates remained unaffected by a 3-week SSRI regimen. However, they did observe a reduction in reinforcement sensitivity following three-week SSRI administration. Overall, these findings underscore the intricate role of serotonin in reinforcement learning, highlighting the nuanced effects of serotonin manipulation on reward and punishment processing. The variability in results across studies underscores the complexity of serotonin’s involvement in cognitive processes and underscores the need for further investigation to elucidate its precise mechanisms.

One potential reason for the mixed results may stem from the different experimental manipulations used. Acute tryptophan depletion is thought to temporarily reduce brain serotonin levels, whereas SSRI administration is generally assumed to increase extracellular serotonin. However, acute SSRI as a single dose can actually decrease serotonin in some brain regions [1]. This complexity arises partly from the potential variations in pre- and post-synaptic actions of these medications. Luo et al. [23] re-examined a number of studies that manipulated serotonin in both rats and humans and while the results show a clear role of serotonin in learning and behavioural flexibility, it does show the complex interactions of the different doses and administration duration [23]. Furthermore, evidence suggests that the neuroplasticity effects of SSRIs might only manifest following more prolonged administration periods, typically spanning 14 to 21 days [24, 25]. Indeed, Johansen et al. [26], using positron emission tomography (PET), have recently shown that neuroplasticity was related to the duration spent on escitalopram [26]. Consequently, chronic SSRI administration may yield more reliable outcomes. Notably, chronic SSRI administration serves as an experimental paradigm closely resembling the treatment regimen for MDD, where clinical improvement is usually only seen after several weeks of treatment.

Reinforcement learning involves a complex interplay among various brain regions. A number of brain regions involved in reinforcement learning, include the ventromedial prefrontal cortex (vmPFC), the anterior cingulate cortex (ACC), and subcortical structures such as the striatum and nucleus accumbens [21, 27–32]. For example, Eldar et al. [27] demonstrated that the function and structure of the striatum were predictive of individual harm avoidance behaviours, emphasizing its role in reinforcement learning processes. Similarly, Niv et al. [32] highlighted the involvement of the nucleus accumbens in risk sensitivity, which plays a crucial role in learning.

Furthermore, additional brain regions such as the orbital frontal cortex and parietal cortex are also implicated in the processing of reward value [33, 34]. Importantly, Guo et al. [34] revealed that uncertain rewards elicited more widespread activation in the brain, indicating the involvement of multiple regions when the reward was probabilistic or uncertain. Serotonin has also been shown to modulate neural mechanisms during reinforcement learning tasks. For example, Scholl et al. [21] found increased reward and effort learning signals in the vmPFC and ACC, respectively, following 2 weeks of SSRI administration. Notably, the rewards and punishments used in their task differed, with rewards being monetary and punishments involving effort, once again highlighting the complexity of serotonin’s influence on reinforcement learning. These findings emphasise the distributed nature of reinforcement learning processes, with multiple brain regions contributing to different aspects of reward and punishment processing, decision-making, and learning. Understanding the interactions among these regions and neurotransmitter systems is crucial for elucidating the neural mechanisms underlying reinforcement learning and related behaviours.

In the present study, we used a double-blind placebo-controlled design to examine the effects of the SSRI escitalopram administered on average for 26 days, on reinforcement learning. In addition, we examine the neural substrates, using task-based functional magnetic resonance imaging (fMRI), that are associated with the effect of escitalopram on reinforcement learning. Escitalopram was chosen for the present study as it shows very high selectivity for the serotonin transporter and is thus the best choice for testing pharmacologic actions of SSRIs [35–37]. Moreover, escitalopram is one of the best-tolerated SSRIs [35–37]. Given the previous literature on serotonin potentially differentially affecting reward and punishment, our reinforcement learning paradigm included both reward and punishment trials. We hypothesised that SSRI treatment would affect reinforcement-related behaviour. In addition, the SSRI treatment would affect the brain regions previously identified as being involved in reinforcement learning. This is the first study to examine the effects of three-week SSRI using a reinforcement learning paradigm.

## Methods

### Participants

This pre-registered study used a double-blind placebo-controlled design with 64 healthy volunteers (Table 1) of whom 32 received 20 mg daily of escitalopram and 32 received placebo for at least 21 days (escitalopram, mean(s.d.)= 26.06(2.78) days; placebo, mean(s.d.)= 26.06(3.34) days; t(60.03) = 0.00; *p* = 1.00; d = 0.00). Participants were semi-randomised (by a staff member not involved with the participants or the data analysis) into the two groups, which were matched for age, sex and intelligence quotient (IQ) (Reynolds Intellectual Screening Test, RIST). Participants aged between 18 and 45 were recruited from an established database of healthy volunteers at the Neurobiology Research Unit at the Copenhagen University Hospital Rigshospitalet. The study was pre-registered on clinicaltrials.gov (NCT04239339). Participants underwent a medical screening prior to enrolment in the study to ensure they were eligible for inclusion. The study was conducted between May 2020 and October 2021. Exclusion criteria are detailed in the Supplementary Material.

**Table 1:** Demographics.

	Placebo (*n* = 32)	Escitalopram (*n* = 32)	t or χ2	p	Cohen’s d or phi
Age	25.38 (5.77)	24.25 (5.56)	t = 0.79	0.43	d = 0.20
Sex	21 Females (65.63%)	21 Females (65.63%)	χ2 = 0.00	1.00	φ = 0.00
IQ	111.50 (9.51)	112.22 (9.30)	t = −0.31	0.76	d = 0.08

### Ethics

The study was approved by the ethics committee for the Capital Region of Copenhagen, Denmark (H-18038352) and written informed consent was obtained from all participants. All methods were performed in accordance with the relevant guidelines and regulations.

### Experimental procedure

After obtaining written informed consent, participants underwent screening for somatic illness, which included a medical examination, blood screening for somatic disease, an electrocardiogram (ECG), and assessment for the presence of psychiatric conditions using the Danish translation version 6.0.0 of the Mini-International Neuropsychiatric Interview (Sheehan et al., 1998). Eligible participants were then semi-randomly assigned to receive either an effective clinical dose of escitalopram (20 mg daily in capsules of 10 mg) or placebo in identical capsules provided by the Capital Region Pharmacy, for a duration of three to five weeks. Both the participants and the investigators involved in data acquisition and analysis were blinded to the intervention type until completion of data analysis.

Blinded medical personnel provided participants with both oral and written instructions on taking escitalopram, including potential side effects. Participants were directed to take 10 mg daily for the initial three days, followed by an increase to 20 mg daily from the fourth day until the last day of examination, coinciding with the neuropsychological testing visit and fMRI scanning session. Before the cognitive visit, participants completed several self-report questionnaires to assess their psychological state. The results of the cognitive and neuropsychiatric analyses are detailed in a separate article [20].

To ensure treatment compliance, the capsule container was examined during visits, and participants provided blood samples both at the halfway point and during the cognitive visit, usually in the morning (see Figure S1). Additionally, participants maintained a daily medication logbook, which was reviewed during the follow-up assessment. Participants were directed to ingest the drug capsule after providing the blood sample to ensure the measurement of steady-state serum escitalopram levels. A medical professional oversaw participant management, maintaining regular contact throughout the study period.

At the end of the study, participants were asked to state whether they believed they had received escitalopram or placebo. Among those in the escitalopram group, 53% accurately guessed that they received escitalopram, while 16.12% of participants in the placebo group thought they received escitalopram. A comparison between the two groups revealed a significant disparity in the ability to correctly discern group membership (χ2 (1, N = 63) = 9.48, *p* = 0.01 [two-tailed]). Thus, the accuracy of guessing the correct allocation in the escitalopram group was at chance level.

### Reinforcement learning paradigm

**Fig. 1:**
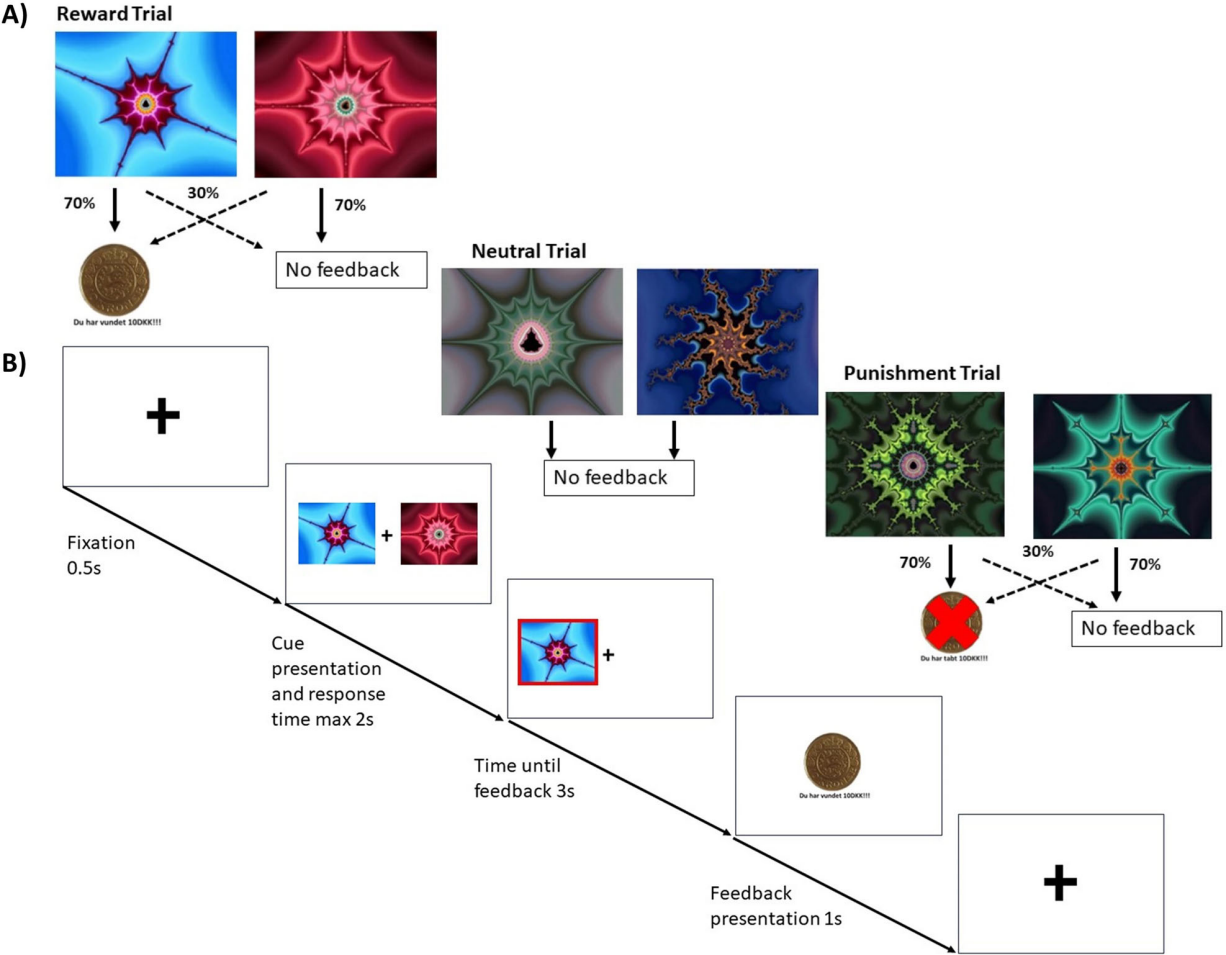
Schematic of the Reward Learning Paradigm. **A** shows the three different trial types and feedback probabilities. **B** demonstrates the timeline of the experimental task. Participants are first presented with a fixation cue for 0.5 s, followed by the presentation cues and they have a maximum of 2 s to respond, their choice is then displayed for 3 s until the feedback is presented for 1 s.

During the fMRI scan, subjects carried out a probabilistic learning paradigm that required making choices to maximize wins and minimize losses (Fig. 1), adapted from previous similar tasks [28–30, 38]. In each trial, one of three possible probabilistic pairs of abstract pictures was randomly presented: a rewarding, punishing, or neutral pair (32 trials of each valence). For each trial, the subject used a button press to indicate a choice of picture (the picture on the right or left). When viewing the probabilistic rewarding pair, selection of one of the pictures led to a financial win with a 70% probability and of a no-change outcome with 30% probability, whereas the selection of the other picture led to gain with only 30% probability. The probabilistic punishing pair led to a financial loss on 70 and 30% trials depending on stimulus choice, and the neutral pair led to no change. The participants completed 3 blocks with new stimuli in each block. All blocks were analysed together to increase the number of trials and thereby, the power in the analyses, particularly the imaging analysis.

### Behavioural analysis

All statistical analyses were conducted in R, version 4.1.1 (R Foundation for Statistical Computing).

The group comparisons for the accuracy and reaction time were conducted using analysis of covariance with both within group (feedback: reward vs punishment) and between group (administration: placebo vs escitalopram) factors controlling for age, sex and IQ. The analysis was conducted using the *aov* function in R. Multiple comparisons correction was conducted using the Benjamini-Hochberg false discovery rate (FDR) with q = 0.05. The *p*-values reported are uncorrected.

To analyse the reinforcement learning paradigm, we used sophisticated computational modelling approach, by fitting families of hierarchical Bayesian reinforcement learning models to trial-by-trial task data [20, 39, 40]. Model comparison was conducted between four models using a bridge sampling estimate of the marginal likelihood using the *bridgesampling* [41] function in R. Model 1 included a reward learning rate, punishment learning rate and reinforcement sensitivity; Model 2 included a reward learning rate, punishment learning rate, reinforcement sensitivity and stimulus stickiness, Model 3 included a combined learning rate, reinforcement sensitivity and stimulus stickiness; and Model 4 used an experience weighted approach [42] which includes learning rate, inverse temperature and experience weight. In Model 4, learning from reinforcement is modulated by an “experience weight” for a stimulus; the experience weight for a stimulus is updated every time it is chosen, and its change over time is governed by a decay factor. In this model, the softmax inverse temperature was also a parameter able to vary.

We analysed the differences in parameter values between groups by first calculating group mean differences (MDs) per parameter. The 90 and 95% highest density intervals (HDIs) of the posterior distribution per MD were then calculated and inspected to check whether they included zero (evidence for no difference between groups). Full details of model formulation, model fitting, and parameter recovery are provided in the Supplementary Material.

### Image acquisition

At the end of the intervention period, participants completed an MRI scan on a 3 T Siemens Magnetom Prisma scanner (Erlangen, Germany) using a 32-channel head coil. We acquired a high-resolution, whole-brain, T1-weighted MPRAGE structural scan (inversion time = 972 ms, repetition time = 2000 ms, echo time = 2.58 ms, flip angle = 8°, in-plane matrix = 256 × 256 mm^2^, in-plane resolution = 0.9 × 0.9 mm^2^, 224 slices, slice thickness = 0.9 mm). We also acquired blood oxygen level-dependent (BOLD) fMRI scans during the reinforcement learning paradigm using a T2*-weighted gradient echo-planar imaging (EPI) sequence (TR = 2000 ms, TE = 30 ms, flip angle = 70°, in-plane matrix = 76 × 76 mm^2^, in-plane Resolution=3 × 3 mm^2^, 35 slices (thickness = 3.0 mm, gap between slices= 0.6 mm). We acquired a gradient field map to minimise spatial distortions in the EPI BOLD fMRI acquisition.

### Image pre-processing

All pre-processing was conducted in SPM 12 (https://www.fil.ion.ucl.ac.uk/spm/software/spm12/). Functional images were slice-timing corrected, realigned and unwarped to correct for head movements and EPI distortions; co-registered and segmented to normalise images into standard space based on the MNI template, for group level analysis; and smoothed with an 8 mm full-width half-maximum (FWHM) Gaussian kernel, to account for residual inter-subject differences. The default SPM12 steps were used. We used the FSL motion outliers’ function to determine the framewise displacement of each image. We determined that participants with mean framewise displacement (FD) > 0.20 mm would be excluded [43]. Movement was small in the cohort and no participants were excluded.

### Neuroimaging analysis

Statistical imaging analysis was also performed in SPM 12. The data from each participant was analysed using general linear models (GLM) and analyses were performed according to an event-related design. The explanatory variables (EVs) that we used were the onset times of the cue stimuli presentation and the feedback stimuli presentation for both reward and punishment trials. They were modelled as 0 s duration events. The 6 realignment parameters were included in the design matrix to correct for signal changes due to head movement. An additional set of harmonic regressors was used to account for any temporal low-pass frequency variance within the data that is typical to fMRI signal with a cut-off of 1/128 Hz. All regressors were convolved with the canonical haemodynamic response function.

In the first-level analysis, the GLMs were used to generate contrast images for our four effects of interest, which included cue presentation and feedback presentation for both reward and punishment conditions. For each contrast, we tested whether the parameter estimates (activation levels) at each voxel were significantly greater than zero.

For the group level analysis to compare between the placebo and escitalopram groups a two-sample t-test was conducted for the reward and punishment trial separately. The analysis was conducted as a whole brain analysis. Voxel-wise results were thresholded at *p* < 0.05, corrected for family-wise error (FWE) at the peak level, to control for multiple comparisons across the entire brain volume as per random field theory in SPM.

## Results

### Demographics

The analysis confirmed that the two groups were well matched and there were no significant differences in age, sex or IQ (see Table 1).

### Behavioural results

**Fig. 2:**
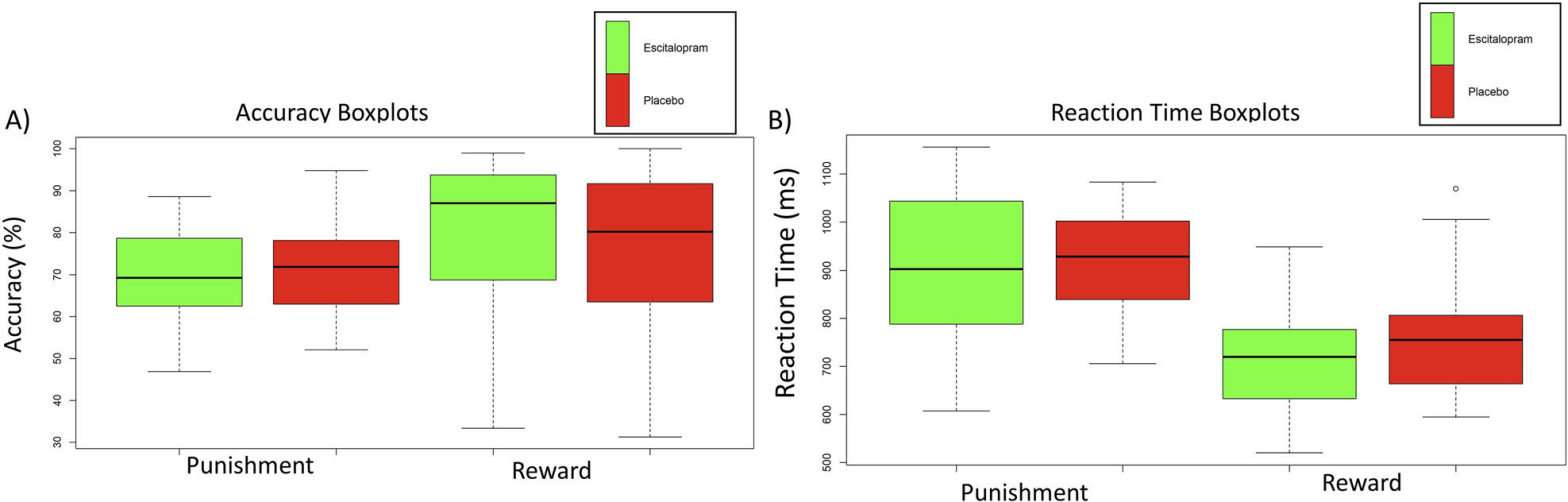
Accuracy and reaction time performance during reward and punishment trials. **A** depicts the accuracy performance, (**B**) depicts the reaction time performance.

There was no main effect of group between the escitalopram and placebo groups for accuracy (F = 0.29, *p* = 0.59, ŋ^2^ < 0.01) or reaction times (F = 1.71, *p* = s.19, ŋ^2^ < 0.01). There was a significant main effect of feedback (reward and punishment) for accuracy (F = 5.83, *p* = 0.16, ŋ^2^ = 0.04) and reaction time (F = 70.67, *p* < 0.001, ŋ^2^ = 0.36). The results showed that accuracy was better in the reward trials than the punishment trials, and that reaction time was significantly faster in reward trials compared to punishment trials. There was no significant interaction effect between group*task for accuracy (F = 0.93, *p* = 0.34, ŋ^2^ < 0.01) or reaction time (F = 0.52, p = 0.47, ŋ^2^ < 0.01). Performance is displayed in Fig. 2.

#### Computational modelling results

**Fig. 3:**
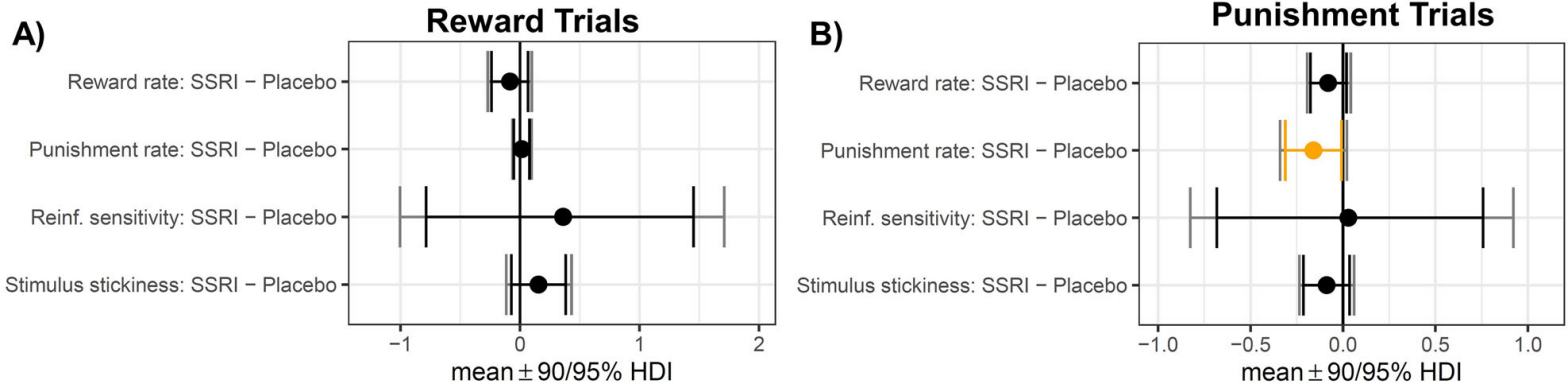
Computational modelling results for the reward and punishment trials. Error bars indicate no credible differences in posterior distributions between placebo and escitalopram groups for which the 90/95% highest density intervals (HDI) excluded 0. **A** depicts the results for the reward trials, (**B**) depicts the results for the punishment trials. Error bars in orange indicate credible differences in posterior distributions between placebo and escitalopram groups for which the 90% highest density interval (HDI) excluded 0.

Hierarchical Bayesian modelling showed that Model 2 was the best model. This revealed that for reward trials, there were no group differences between the escitalopram and the placebo group at the credible difference level of 95% for any of the four model parameters: reward learning (mean difference (MD) = −0.08 [95% HDI −0.27 to 0.10, punishment learning (MD = 0.02 [95% HDI −0.06 to 0.10]), stimulus stickiness (MD= 0.16 [95% HDI −0.11 to 0.43]) or reinforcement sensitivity (MD= 0.36 [95% HDI −1.00 to 1.71]). The results are represented in Fig. 3 and the model comparison is presented in Table S1.

For the punishment trials, the escitalopram group had lower punishment learning rate than the placebo group at the credible difference level of 90% (MD = −0.15 [90% HDI −0.31 to −0.01). There were no differences between the groups for any of the other three model parameters: reward learning (MD = −0.08 [95% HDI −0.19 to 0.04]), stimulus stickiness (MD = −0.08 [95% HDI −0.24 to 0.06]) or reinforcement sensitivity (MD= 0.02 [95% HDI −0.83 to 0.92]). The results are represented in Fig. 3 and the model comparison is presented in Table S2.

### Neuroimaging results

**Fig. 4:**
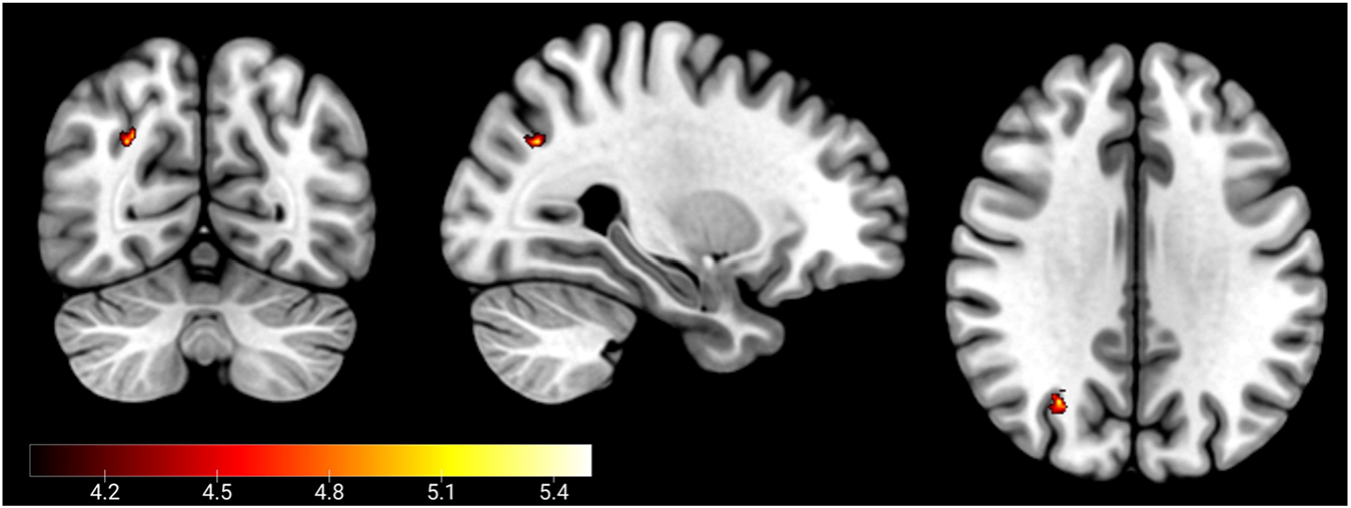
Decreased BOLD activation in the escitalopram group vs the placebo group during reward trials. MNI Coordinates (28, −66, 32). *p* < 0.05 peak-level FWE corrected.

The neuroimaging results showed no group differences for cue presentation for either the reward or punishment trials. For feedback, the escitalopram group had reduced activation in the intraparietal sulcus compared to the placebo group during the reward trials. There were no differences for feedback on punishment trials. The results are shown in Table 2 and Fig. 4.

**Table 2:** Reduced BOLD activation in the escitalopram group compared to the placebo group for feedback on reward trials.

Brain Region	BA	x	y	z	z-value	Cohen’s d
Intraparietal Sulcus	19	28	−66	32	5.03	0.63

*BA* Brodmann’s Area.

## Discussion

In this double-blind, placebo-controlled study, a relatively large group of healthy volunteers received either escitalopram or placebo for an average of 26 days. This is the first study to examine the effects of three-week SSRI using a reinforcement learning paradigm. The key findings were that the escitalopram group had a lower punishment learning rate when compared to the placebo group during punishment trials. In addition, the escitalopram group showed reduced activation in the intraparietal sulcus compared to the placebo group during reward trials.

### Behavioural results

Using the standard analysis of covariance accuracy and reaction time, results showed no main effect of group, suggesting no differences between the escitalopram and placebo groups for either reward or punishment trials. There was a significant main effect of feedback demonstrating that accuracy was decreased, and reaction time was increased in punishment trials compared to reward trials. There was no interaction between feedback and group, suggesting that the placebo and escitalopram group both performed worse in the punishment compared to reward trials. These results together suggest that participants find it more difficult to learn from punishment than from reward, but that escitalopram does not have an effect on accuracy or reaction time.

For the more sophisticated computational modelling, our results showed that the escitalopram group had a lower punishment learning rate during punishment trials compared to placebo controls. There were no group differences for learning during reward trials. In addition, there were no group differences for reinforcement sensitivity or stimulus stickiness between groups for reward or punishment trials. Our results suggest that escitalopram has a greater effect on learning from punishment than learning from reward. Previous studies have shown a similar asymmetry where serotonin affects learning from punishment to a greater extent [13, 19, 22, 44]. The results from the present study suggest that three-week escitalopram impairs punishment learning, but reward learning remains stable. It is worth noting that these studies differ not only in the exact reinforcement learning task used, but also the serotonin manipulation used. The present study and that of Michely et al. [19] for example differ in the dosage and length of SSRI administration. In our study, we used at least 3 weeks of SSRI treatment, whereas Michely et al. [19] used only one week. Given the findings that the neuroplasticity effects can take 14–35 days [24–26], this may be one possible reason for the difference in findings. For this reason, in studies of chronic SSRI effects, the duration should be at least 21 days. In addition, given the importance of learning from reinforcing feedback in everyday life and the large number of individuals on SSRI treatment it is crucial to understand the effects of chronic SSRI to optimise treatment and quality of life for patients with neuropsychiatric conditions. Moreover, understanding the neural mechanisms through which serotonin exerts its effects on affect, cognition and behaviour is of importance to reduce the severity of clinical symptoms.

### Neuroimaging results

Our fMRI results showed a key novel finding namely that there was decreased activation in the intraparietal sulcus in the escitalopram group compared to the placebo group during performance on reward trials. The same region showed decreased activation during punishment trials, but this did not survive the peak-level FWE correction. There were no increased regions of activation in the escitalopram group when compared to the placebo group. Several studies have implicated the intraparietal sulcus in reward processing. Specifically it has been shown to play a role in both the decision-making during uncertainty [34, 45–47], as well as being involved in a ‘value-driven attention network’ where attentional resources are allocated to valuable choices [48–51].

For example, it has been demonstrated that neurons in the parietal cortex of non-human primates encode the probability of rewards, particularly through eye-movements, suggesting its involvement in reward-based decision-making processes [45]. Neuroimaging studies in humans have also implicated the intraparietal sulcus in reward processing. One fMRI study showed increased activity in the parietal cortex during probabilistic reward anticipation tasks, thereby suggesting a role in both the anticipation of rewarding outcomes, but also during uncertainty [46], as well as when the reward is uncertain [34]. A further fMRI study has shown that the IPS is associated with state prediction error which is a measure the degree of surprise in encountering a new state, based on the subjects’ current understanding of the probabilities that link one state to another following specific actions [47]. Therefore, our finding of decreased IPS activation in the escitalopram group potentially reflect an aberrant response to the probabilistic and uncertain elements of the task. This pattern could imply that escitalopram affects neural mechanisms involved in processing state prediction errors or adapting to probabilistic task demands, thereby impacting decision-making under uncertainty.

In terms of the involvement of the intraparietal sulcus in a ‘value-driven attention network’, it has been shown in non-human primates that the intraparietal sulcus responds to rewards during visual attention tasks, indicating its role in integrating reward-related information with attentional processes [48]. Similarly, it has been shown to respond preferentially to stimuli that predict available reward [51]. In humans, the intraparietal sulcus has been shown to bias attentional resources to stimuli associated with reward [49]. Our finding of reduced learning from punishment in the escitalopram group may be due to the fact that escitalopram disrupts the encoding of the value of reinforcing feedback. These results make sense when considering that SSRIs are used to treat the negative bias and responses to abnormal feedback in MDD. It may be that SSRIs blunt reinforcement encoding, and in fact blunted affect has often been reported by patients [52, 53] and previously demonstrated in our own research [20]. This may lead to reduced sensitivity to reinforcers, which thereby impacts adaptive learning from feedback. This is in line previous findings of disrupted reinforcement sensitivity and learning following serotonin manipulations [17, 18, 20, 23]. However, this may be an advantage in MDD as it reduces the abnormal response to negative feedback.

Taken together, the involvement of the intraparietal sulcus in reward processing and the impact of serotonin on reinforcement learning provide a plausible framework for understanding the observed differences in intraparietal sulcus activation between the placebo and escitalopram groups. The interplay between serotonin, reward processing, and the intraparietal sulcus highlights the complex neurobiological mechanisms underlying the modulation of reward-related behaviours.

We did not see differences between the groups in more traditional brain regions associated with reward, for example the nucleus accumbens and the anterior cingulate cortex. However, many of these regions are involved in neuroimaging studies examining the role of dopamine not the role of serotonin. Studies have also demonstrated that when outcomes are uncertain there is a much more widespread pattern of activation in the brain [34]. The outcomes in the reinforcement paradigm used in the present study were indeed uncertain. One previous study of the effects of citalopram on reinforcement learning showed enhanced reward and effort learning signals in a widespread network of brain regions, including ventromedial prefrontal and anterior cingulate cortex [21]. In addition, we did not see group differences in neuroimaging prediction error signals (see Supplementary Material). However, as we have noted in the mixed behavioural findings, the specific manipulations and durations of serotonin seem to affect the results. Further research which examines the chronic effects of serotonin is needed in both healthy volunteers and patients with MDD. This is particularly important considering that some national guidelines, for example the National Institute for Health and Care Excellence (NICE) in the UK and the American Psychological Association (APA) in the USA, suggest that if there is no treatment response to increase the dosage or even change the drug, after 4–6 weeks [54, 55]. Given that SSRIs are administered chronically to patients with neuropsychiatric disorders, the present findings hold more clinical relevance than acute studies.

## Conclusion

In this double-blind placebo-controlled design of escitalopram administered on average for 26 days to healthy individuals, we showed that there was reduced learning from punishment in the escitalopram group. Using fMRI, our key and novel finding was that there was reduced activation in the intraparietal sulcus in the escitalopram group during reward trials compared to the placebo group. Given the role of the intraparietal sulcus in reinforcement learning, specifically in uncertainty and outcome value it may be that reinforcement learning is altered by serotonin through its effects on encoding of value outcomes. This may lead to reduced sensitivity to reinforcers, which thereby impacts adaptive learning from feedback. In addition, it seems that escitalopram specifically disrupts the processing of probabilistic feedback. These novel findings provide strong evidence for a key role of serotonin and the intraparietal sulcus in reinforcement learning. Given the importance of learning from reinforcing feedback in everyday life and the large number of patients on SSRI treatment it is crucial to understand the effects of chronic SSRIs to optimise treatment and quality of life for patients with neuropsychiatric conditions. Moreover, understanding the neural mechanisms through which serotonin exerts its effects on affect, cognition and behaviour is of importance for clinical treatment of MDD.

## Supplementary information

Supplementary Material

## Data Availability

Applications to use data from the CIMBI database can be made to the Neurobiology Research Unit, Rigshospitalet, Copenhagen University Hospital.

## Code availability

The code supporting the modelling analyses are available at https://github.com/RudolfCardinal/rlib.
