# Supplementary material for: Computational modelling and neural correlates of reinforcement learning following three-week escitalopram: a double-blind, placebo-controlled semi-randomised study
Source: Transl Psychiatry. 2025 May 21;15:175. doi: 10.1038/s41398-025-03392-6 (PMC12095678; doi:10.1038/s41398-025-03392-6)
Supplement: Supplementary file 1 — Supplementary Material [file 41398_2025_3392_MOESM1_ESM.docx]

# Computational modelling and neural correlates of reinforcement learning following three-week escitalopram: A double-blind, placebo-controlled semi-randomised study.

Christelle Langley^1,2*^, Graham K. Murray^1,3^, Sophia Armand^4,5^, Franziska Knolle^1,6^, Rudolf N. Cardinal^1,3^, Annette Johansen^4^, Peter S. Jensen^4^, Jianfeng Feng^7,8^, Dea S. Stenbæk^4,5^, Gitte M. Knudsen^4,9^, Patrick M. Fisher^4,10^, Barbara J. Sahakian^1,2^

Affiliations:

1. Department of Psychiatry, University of Cambridge, Cambridge, UK
2. Behavioural and Clinical Neuroscience Institute, University of Cambridge, Cambridge, UK
3. Cambridgeshire and Peterborough NHS Trust, Cambridge, UK
4. Neurobiology Research Unit, Copenhagen University Hospital Rigshospitalet, Copenhagen, Denmark
5. Department of Psychology, University of Copenhagen, Copenhagen, Denmark
6. Department of Diagnostic and Interventional Neuroradiology, Technical University of Munich, Munich, Germany.
7. Institute of Science and Technology for Brain-Inspired Intelligence, Fudan University, Shanghai, China.
8. Department of Computer Science, University of Warwick, Coventry, United Kingdom.
9. Department of Clinical Medicine, University of Copenhagen, Copenhagen, Denmark
10. Department of Drug Design and Pharmacology, University of Copenhagen, Denmark

*Corresponding Author:

Christelle Langley, Herchel Smith Building for Brain and Mind Sciences, Forvie Site, Robinson Way, Cambridge, CB20SZ, [cl798@medschl.cam.ac.uk](mailto:cl798@medschl.cam.ac.uk)

# Supplementary Material:

## Supplementary Methods:

### Participants:

The full exclusion criteria were as follows:

1. Current or former primary psychiatric disorder as classified in DSM-V or WHO ICD-11
2. Current or former neurological disease or severe somatic disease
3. History of head injury or concussion resulting in loss of consciousness for more than two minutes
4. Current use of psychoactive medication
5. Drug use other than tobacco and alcohol within the last 30 days
6. Current alcohol or drug abuse
7. Use of cannabis more than 50 times
8. Use of illegal psychoactive drugs more than 10 times for any drug
9. Current use of any drugs likely to influence the test results
10. Nicotine addiction
11. Allergy to the ingredients in the administered drug
12. Abnormal ECG such as prolonged QT syndrome
13. Dizzy when changing from supine to upright position (e.g. postural orthostatic tachycardia syndrome)
14. Mild hypotension (blood pressure below 100/70 mmHg) or hypertension (blood pressure above 140/90 mmHg)
15. Contraindications for MRI such as a pacemaker or other metal implants
16. Pregnancy or lactation
17. Current or past learning disability
18. Non-fluent in Danish
19. Pronounced visual or auditory impairments
20. Severe physical impairments affecting eyesight or motor performance.

**Participants:**

### Escitalopram was well-tolerated in this study, as there were no significant group differences in dropout rates due to side effects between the placebo and escitalopram groups (χ2= 1.68, p = .10 [one-tailed]).

### Hierarchical Bayesian Modelling:

To investigate processes underlying learning and decision-making on the probabilistic reversal learning task (PRL), a family of simple reinforcement learning (RL) models were fitted to data. Modelling first involves formulating a mathematical function equipped with different parameters of interest to analyse trial-by-trial data. Model code was adapted from prior studies (Kanen et al., 2019; Langley et al., 2023; Marzuki et al., 2021).

Model 1 included three parameters: a reward learning rate parameter (*α*_rew_), a punishment learning rate parameter (*α*_pun_) and a reinforcement sensitivity parameter (*τ_reinf_*). A value (*Q*) was assigned to each task stimulus, representing the expected reward associated with them. A high *Q* denoted a higher chance of reward associated with a stimulus, while a lower *Q* indicated a lower chance of a reward. *Q_k_* (the value for stimulus *k*) was updated on a trial-by-trial basis via prediction errors that represent the difference between the expected outcome on that trial *t*, *Q_k,t_*, and the actual outcome, *R_t_*. For example, if the expected outcome for a stimulus is 0, and selecting the stimulus on a given trial results in a reward, *Q* for that specific stimulus would increase. Larger prediction errors lead to faster updating of *Q*. On every trial (t), the value of a learning rate parameter (*α*_rew_ or *α*_pun_) determined the extent to which *Q* was adjusted according to the prediction error. Concretely, this was done according to the Rescorla–Wagner rule:

*Q_k_*_,_*_t_*_+1_ = *Q_k_*_,_*_t_* + *α*_rew_(*R_t_* – *Q_k_*_,_*_t_*), if *R_t_* = 1;

*Q_k_*_,_*_t_*_+1_ = *Q_k_*_,_*_t_* + *α*_pun_(*R_t_* – *Q_k_*_,_*_t_*), if R*_t_* = 0.

where *k* represents a specific stimulus (stimulus 1 or 2) and *t* represents the current trial. *R* was 1 following a rewarded outcome, and 0 following an unrewarded outcome. The term *R_t_* – *Q_k_*_,_*_t_* is the prediction error.

*α* values varied between 0 and 1. Values of *α*_rew_ govern sensitivity to (learning from) positive prediction errors (i.e., rewarding trials) while *α*_pun_ governs learning from negative prediction errors (punishing outcomes); the use of two learning rates allows the investigation of valence-specific learning.

Finally, *τ_reinf_* (reinforcement sensitivity) is an inverse temperature parameter, used within a softmax function to determine the probability *p* of choosing a stimulus *k* on trial *t*:

$$p_{k,t}=\frac{exp\left( \tau_{reinf}Q_{k,t} \right)}{\sum_{i=1}^{n} exp\left( \tau_{reinf}Q_{i,t} \right)}$$

*τ_reinf_* determined the extent to which participants’ actions were driven by *Q* values associated with the chosen stimulus. A high *τ_reinf_* leads to more “exploitative” behaviour, whereby a participant chooses mostly to maximise their rewards (i.e., participants strongly prefer the choice with the higher *Q* value). A low *τ_reinf_* enables more exploratory behaviour (lesser preference for the choice associated with the higher *Q* value).

*Model 2*

Model 2 was identical to Model 1 but with the addition of *τ*_stim_ (stimulus stickiness), which is an inverse temperature parameter that reflects the tendency for a participant to respond to the same stimulus chosen in a previous trial regardless of feedback received. Greater values of *τ*_stim_ denote increased tendency to ‘stick’ with a choice, while low values represent a tendency to switch away from the choice. Thus, *τ*_stim_ enabled us to account for perseverative behaviour. This parameter was added to the softmax function as follows:

$$p_{k,t}=\frac{exp\left( \tau_{reinf}Q_{k,t}+\tau_{stim}S_{k,t} \right)}{\sum_{i=1}^{n} exp\left( \tau_{reinf}Q_{i,t}+\tau_{stim}S_{i,t} \right)}$$

*S* represents whether the stimulus being considered on the current trial (*S_i_*_,_*_t_*) was the same as the one chosen on the previous trial (*S* = 1 for such a repeated choice, 0 otherwise). Thus, this model contained four parameters in total: *α*_rew_, *α*_pun_, *τ_reinf_*, and *τ*_stim_.

*Model 3*

Model 3 was as Model 1 but with only three parameters (*α*, *τ_reinf_*, *τ*_stim_), using a single learning rate for reinforcement (whether rewarded or unrewarded).

*Model 4*

Model 4 was distinct from the models described thus far. It was an experience-weighted attraction (EWA) model used previously (Den Ouden et al., 2013). It contains three free parameters: *φ* (phi), *ρ* (rho), and *β* (beta). The model served to decouple acquisition (pre-reversal) and reversal via the experience decay factor parameter *ρ* that enables the balance between previous experience and new information to tip increasingly in favour of past experiences. The ‘experience weight’ (*n_c_*_,_*_t_*) of a current choice, *c*, reflects how often a stimulus has been chosen. It is updated according to *ρ*:

*n_c_*_,_*_t_* ← *n_c_*_,_*_t_*_–1_ *ρ* + 1

The intuition behind *ρ* is that over time, experience accumulated during acquisition could make reversal more difficult, leading to perseveration. *ρ* was allowed to range between 0 and 1. When *ρ* = 0, predictions are always driven by most recent outcomes, whereas when *ρ* = 1 all trials are weighted equally, leading to perseveration of responses after reversal. The value function of a choice on every trial, *v_c_*_,_*_t_* (similar to *Q_t_*), is updated according to the outcome (rewarded or unrewarded), *λ*, and the pay-off decay factor *φ*, which is equivalent to the learning rate in Model 1.

*v_c_*_,_*_t_* ← (*v_c_*_,_*_t_*_–1_ *φ* *n_c_*_,_*_t_*_–1_ + *λ_t_*_–1_) / *n_c_*_,_*_t_*

When *ρ* = 0, *n_c_*_,_*_t_* on every trial becomes 1 and therefore reduces to a standard Rescorla–Wagner model. Equivalent to models described earlier, the probability *P* of choices *c* were determined via a softmax function:

$$P\left( c_{t+1}=k \right)=\frac{e^{{\beta V}_{c=k,t+1}}}{\sum_{i=1}^{n} e^{{\beta V}_{c=i,t+1}}}$$

where, as before, the inverse temperature parameter *β* controls the extent to which choices are made according to the value function *V*. Low values of *β* lead to more exploratory choices, while high values lead to choices that serve to maximise rewards.

**Model Fitting and Parameter Estimation**

Models were fit to trial-by-trial data using a hierarchical Bayesian approach, estimating the posterior distribution of the model parameters at the individual subject and group levels. This enabled estimating parameter distributions per group while controlling for inter-subject variability in behaviour. At the top of the hierarchy, separate distributions were defined for placebo and escitalopram groups. Parameter estimations for each group were sampled from the following prior distributions using exactly the same priors for each group:

*α*_group_, *α*_group,rew_, *α*_group,pun_, *φ*_group_, *ρ*_group_ ~ Beta(1.2, 1.2)

*τ*_group_, *β*_group_ ~ Gamma(4.82, 0.88)

*τ*_group,stim_ ~ Normal(0, 1)

Inter-subject variability, σ, was sampled from half-normal prior distributions, constrained to be positive:

*σ_α_*, *σ_α_*_rew_, *σ_α_*_pun_, *σ_τ_*_stim_, *σ_φ_*, *σ_ρ_* ~ half-Normal(0, 0.05)

*σ_τreinf_*, *σ_β_* ~ half-Normal(0, 1)

Subject-level parameters were sampled from normal distributions whose means were the group level parameter values and whose standard deviations were the inter-subject variability parameter values (restricted to the valid range for the parameter). For example, in the case of *α*_rew_:

*α*_rew,subject_ ~ [N(*α*_rew,group(subject),_ *σ_α_*_rew)_$]_{0}^{1}$

All priors were obtained from earlier studies for model 1: α_rew_ (den Ouden et al. (2013)), α_pun_ (den Ouden et al. (2013)), τ_reinf_ (Gershman (2016)); model 2: α_rew_ (den Ouden et al. (2013)), α_pun_ (den Ouden et al. (2013)), τ_reinf_ (Gershman (2016)), τ_stim_ (Christakou et al. (2013)); model 3: α (den Ouden et al. (2013)), τ_reinf_ (Gershman (2016)), τ_stim_ (Christakou et al. (2013)); and model 4: ρ (den Ouden et al. (2013)), φ (den Ouden et al. (2013)), β (Gershman (2016)). The intersubject deviations for τ_reinf_, β are also based on Gershman (2016).

All models were fitted to data using Markov chain Monte Carlo (MCMC) sampling implemented in RStan v2.21.2. Eight randomly initialised MCMC chains were used. Convergence of chains was confirmed using the potential scale reduction statistic R̂. A cut-off R̂ value of 1.2 (Kanen et al., 2019; Langley et al., 2023; Marzuki et al., 2021) was used to check that the chains were well mixed for each parameter.

#### Model Comparisons

Models for both tasks were compared using bridge sampling via the “bridgesampling” R package (Gronau et al., 2017). This method enables selection of the best-fitting model by accounting for the prior probability and marginal likelihood of each model (the likelihood of the data given a specific model). The marginal likelihood is calculated via an estimate of the product of the likelihood of the data given a fitted model and the probability of parameters given the model, integrated over the parameters, which penalises over-complex models and guards against overfitting.

#### Group Differences

Posterior distributions of parameters from the winning model was interpreted using the 95% and 90% highest posterior density intervals (HDIs), also known as the Bayesian credible interval. Parameter comparisons between groups were calculated by examining the difference between the relevant placebo group’s parameter and the corresponding escitalopram group’s parameters (escitalopram – placebo), i.e. to estimate the group mean difference per parameter. The 95% and 90% HDIs of the posterior distribution for the group mean differences were calculated and inspected to check whether they included zero (indicating no credible difference between groups at this level). Multiple comparisons corrections were not applied since they are not necessary for these Bayesian techniques (Gelman et al., 2012; Kruschke, 2010).

**Parameter Recovery for Winning Model**

We conducted parameter recovery to verify the validity of the winning model. Given the computational intensity of the Bayesian method used in our analysis, a full combinatorial exploration of the parameter space was not feasible (Kanen et al., 2019). To address this, we simulated five values per parameter, covering a range that exceeded the posterior group mean values obtained from the actual dataset. For each simulation, all parameters except one were fixed at their central value, allowing only one parameter to vary at a time, as detailed in Table S3. Each run involved simulating 50 identical virtual subjects within a single virtual group performing a simulated behavioural task, with 96 trials and one discrimination per subject. The simulated data were analysed following the same procedures used for the real data, with the exception of excluding intersubject variability measurements (since intersubject variability was zero in this setup). For each parameter, we calculated the mean and 95% highest density interval (HDI) of its posterior distribution. The results are presented in Table S3.

**Simulation of Behavioural Data for Winning Model**

### We simulated behaviour using the posterior group mean parameters from the winning model, with 50 simulated subjects per group (placebo and escitalopram) and no intersubject variability (Kanen et al., 2019), for comparison with the actual behavioural data. The simulation framework used 96 trials with 1 discrimination per subject. We then analysed task accuracy across the simulations.

### Neuroimaging Analysis:

At the first-level analysis we also estimated reward and punishment prediction errors based on the trial-by-trial computational model estimates of reward cue values and prediction errors. These were also entered into a general linear model (GLM) analysis to determine whether neural activation was associated with prediction errors. For the group level analysis to compare between the placebo and escitalopram groups a two-sample t-test was conducted for the reward and punishment prediction errors separately. The analysis was conducted as a whole-brain analysis. Voxel-wise results were thresholded at p<.05, corrected for family-wise error (FWE) at the peak level, to control for multiple comparisons across the entire brain volume at per random field theory in SPM.

## Supplementary Results:

### Biochemical Analysis:


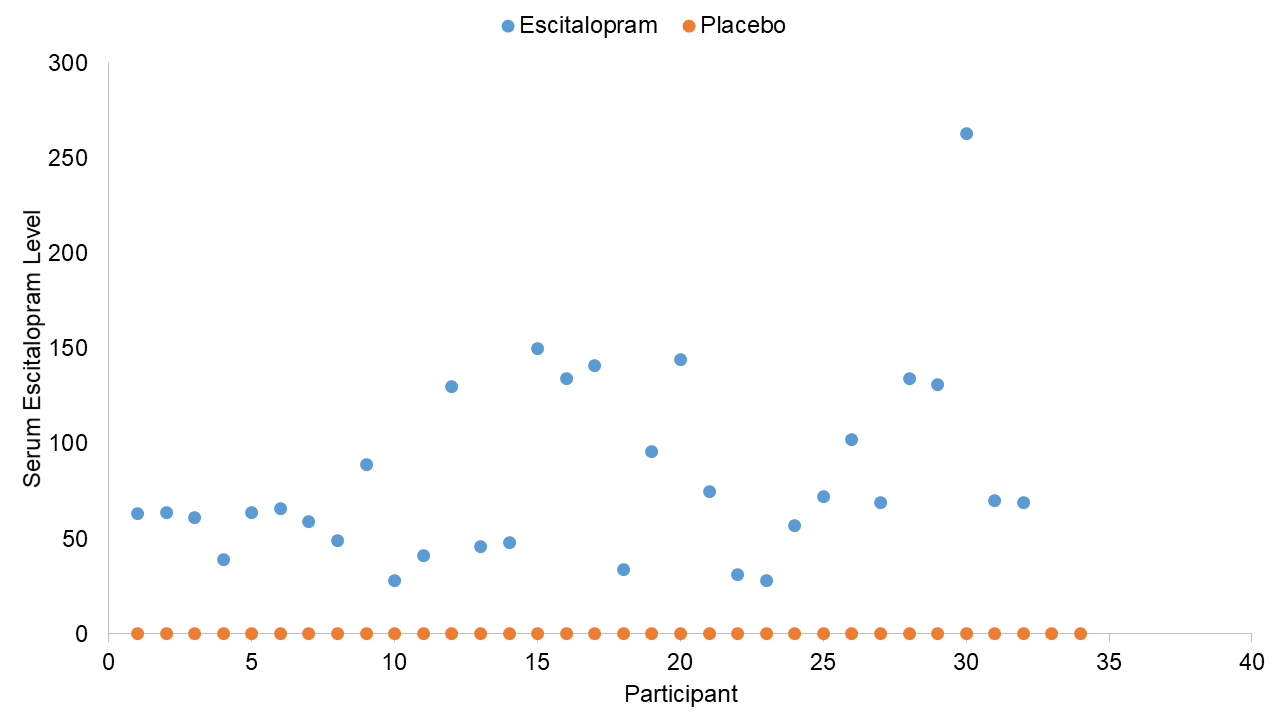


***Figure S1.*** *Serum escitalopram levels in nmol/L at imaging visit.*

### Behavioural results:


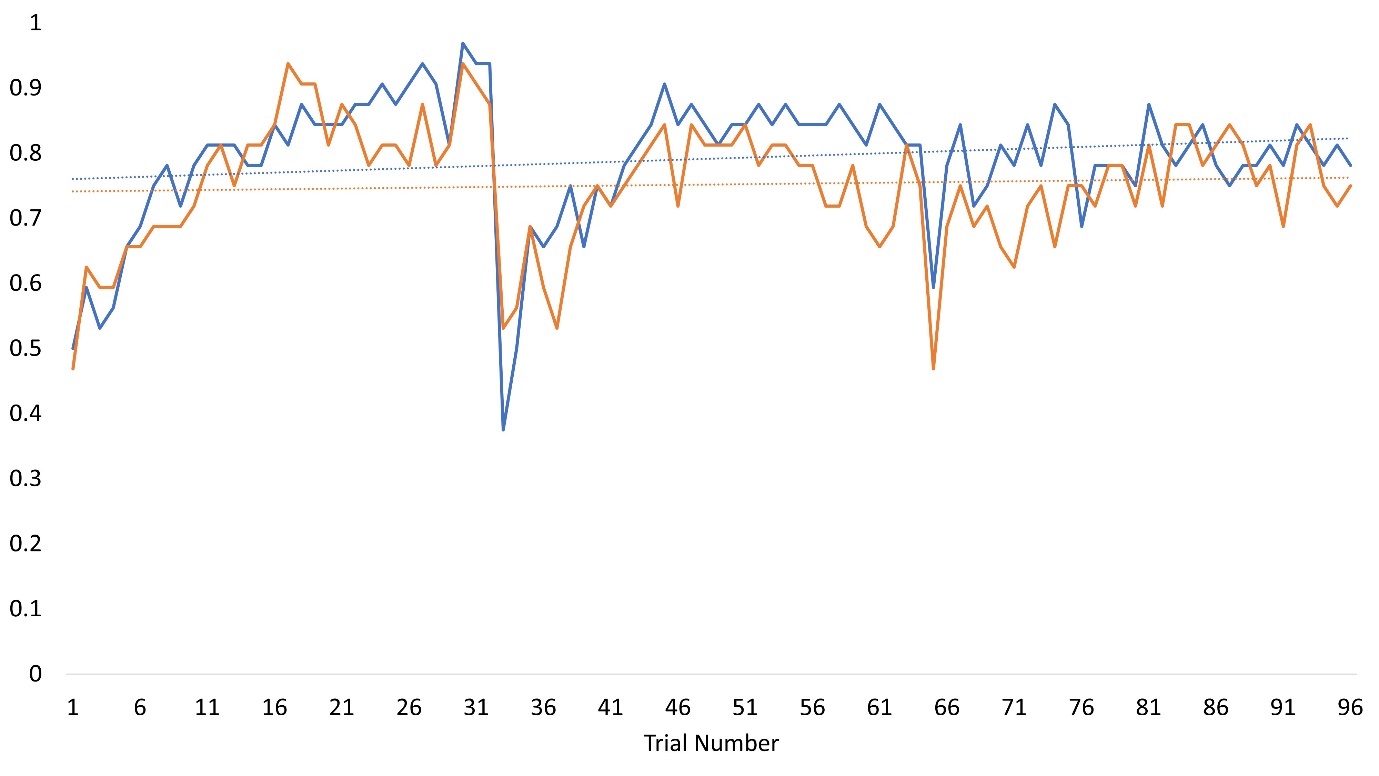


**Figure S2.** Learning Curves for Reward Trials. The *escitalopram group are displayed in blue, and the placebo group in orange.*


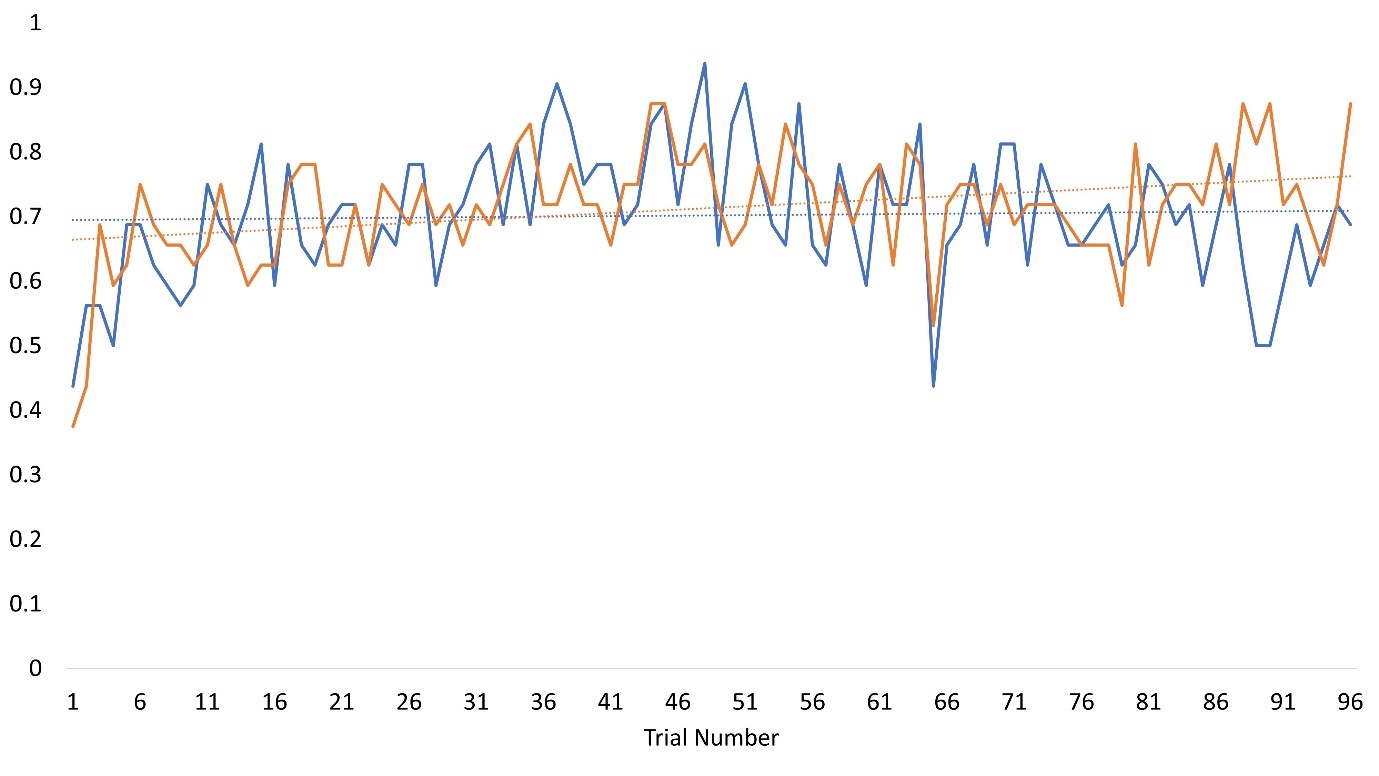


**Figure S3.** Learning Curves for Punishment Trials. *The escitalopram group are displayed in blue, and the placebo group in orange.*

We also tested whether the belief status of the intervention they received within the escitalopram group affected the results. For both accuracy and reaction time there were no differences between participants that guessed placebo or escitalopram for either reward (accuracy: M_placebo_= 78.61(21.46), M_escitalopram_= 79.66(15.69), t(30)= -.16, p= .88, d= .06; reaction time: M_placebo_= 703.94(103.44), M_escitalopram_= 721.66(107.43), t(30)= -.47, p= .64, d= .17) or punishment trials (accuracy: M_placebo_= 72.22(10.73), M_escitalopram_= 68.44(9.60), t(30)= 1.05, p= .31, d= .37; reaction time: M_placebo_= 909.42(131.03), M_escitalopram_= 900.35(166.67), t(30)= .17, p= .87, d= .06).

### Computational modelling results:

Similarly, the belief status of the intervention they received within the escitalopram group did not affect the modelling parameters for either reward (mean difference (MD)= .14 [95% HDI -.03 to .30), stimulus stickiness (MD= .33 [95% HDI -.03 to .70]) or reinforcement sensitivity (MD= -.34 [95% HDI -2.06 to 1.36]) or punishment trials (reward learning rate (MD= -.02 [95% HDI -.15 to ,12]), punishment learning rate (MD= -.01 [95% HDI -.25 to .22), stimulus stickiness (MD= -.10 [95% -.29 HDI to .09 ]) or reinforcement sensitivity (MD= .65 [95% HDI -.48 to 1.83]).

To test the validity of the intersubject standard deviations for α_rew_, α_pun_, α, τ_stim_, ρ, φ as used in Kanen et al. (2019), Langley et al. (2023) and Marzuki et al. (2021), we widened the priors and found the same pattern of results for reward trials (reward learning rate (MD= -.10 [95% HDI -.33 to .12, punishment learning rate (MD= .02 [95% HDI -.07 to .12]), stimulus stickiness (MD= .13 [95% HDI -.23 to .48]) or reinforcement sensitivity (MD= .78 [95% HDI -0.91 to 2.63])) and punishment trials (reward learning rate (MD= -.05 [95% HDI -.17 to .07]), punishment learning rate (MD= -.17 [90% HDI -.36 to -.01), stimulus stickiness (MD= -.08 [95% HDI -.25 to .09]) or reinforcement sensitivity (MD= -.09 [95% HDI -1.16 to .90]).

***Table S1.*** *Model Comparison for the reward trials.*

| Model | Model Rank | Parameters | Log Marginal Likelihood | Maximum R-hat |
| --- | --- | --- | --- | --- |
| 1 | 4 | α_rew_, α_pun_, τ_reinf_ | -691.10 | 1.0016984 |
| 2 | 1 | α_rew_, α_pun_, τ_reinf_, τ_stim_ | -648.85 | 1.0036534 |
| 3 | 2 | α, τ_reinf_, τ_stim_ | -677.38 | 1.002091 |
| 4 | 3 | ρ, φ, β | -687.09 | Poor Convergence |

***Table S2.*** *Model Comparison for the punishment trials.*

| Model | Model Rank | Parameters | Log Marginal Likelihood | Maximum R-hat |
| --- | --- | --- | --- | --- |
| 1 | 2 | α_rew_, α_pun_, τ_reinf_ | -1150.75 | 1.003531 |
| 2 | 1 | α_rew_, α_pun_, τ_reinf_, τ_stim_ | -1147.51 | 1.003658 |
| 3 | 3 | α, τ_reinf_, τ_stim_ | -1152.46 | 1.006273 |
| 4 | 4 | ρ, φ, β | -1158.33 | Poor Convergence |

***Table S3.*** *Parameter recovery for the winning model from simulated data (see Supplementary Methods). Virtual subjects (n = 50) were simulated for each row, using the “true” parameters shown, and the parameters estimated (recovered) from the resulting data via a hierarchical Bayesian model as described in the text. Bold highlights parameter variation (which extended beyond the range of the posterior group means from the placebo/escitalopram groups) and italics the group with all-central values. Recovered parameters are shown in the format “μ [a, b] (R=^R)” where μ is the posterior mean, [a, b] is the 95% HDI, and ^R is the potential scale factor reduction measure of convergence. * indicates values for which the parameters were not correctly recovered as judged by the 95% HDI (occurring in 3 out of 68 tests, or 4.4%, versus an expectation of 5% for 95% HDIs).*

| Process | RL | | | | Reinforcement Sensitivity | | Stimulus Stickiness | |
| --- | --- | --- | --- | --- | --- | --- | --- | --- |
| Parameter | α_rew_ | | α_pun_ | | τ_reinf_ | | τ_stim_ | |
| Sim# | TRUE | Recovered | TRUE | Recovered | TRUE | Recovered | TRUE | Recovered |
| 1 | **0.1** | 0.114 [0.083,0.144] (R=1.001) | 0.35 | 0.387 [0.335, 0.437] (R=1.000) | 3.5 | 3.236 [2.734, 3.727] (R=1.001) | 0.9 | 0.917 [0.854, 0.981] (R=1.001) |
| 2 | **0.2** | 0.205 [0.162, 0.248] (R=1.001) | 0.35 | 0.369 [0.321, 0.415] (R=1.000) | 3.5 | 3.432 [3.085, 3.788] (R=1.001) | 0.9 | 0.902 [0.824, 0.971] (R=1.000) |
| 3 | *0.3* | *0.252 [0.205, 0.300] (R=1.000)** | *0.35* | *0.350 [0.305, 0.397] (R=1.000)* | *3.5* | *3.570 [3.256, 3.877] (R=1.000)* | *0.9* | *0.905 [0.827, 0.980] (R=1.001)* |
| 4 | **0.4** | 0.362 [0.301, 0.424] (R=1.000) | 0.35 | 0.353 [0.309, 0.397] (R=1.000) | 3.5 | 3.626 [3.350, 3.881] (R=1.000) | 0.9 | 0.883 [0.804, 0.967] (R=1.000) |
| 5 | **0.5** | 0.514 [0.442, 0.588] (R=1.000) | 0.35 | 0.355 [0.310, 0.440] (R=1.000) | 3.5 | 3.455 [3.216, 3.696] (R=1.001) | 0.9 | 0.854 [0.764, 0.938] (R=1.000) |
| 6 | 0.3 | 0.323 [0.276, 0.367] (R=1.000) | **0.003** | 0.002 [<0.001, 0.004] (R=1.000) | 3.5 | 3.606 [3.314, 3.912] (R=1.000) | 0.9 | 0.913 [0.847, 0.976] (R=1.000) |
| 7 | 0.3 | 0.258 [0.207, 0.308] (R=1.001) | **0.175** | 0.173 [0.138, 0.207] (R=1.001) | 3.5 | 3.525 [3.258, 3.782] (R=1.001) | 0.9 | 0.912 [0.831, 0.990] (R=1.000) |
| 8 | 0.3 | 0.282 [0.228, 0.340] (R=1.000) | **0.525** | 0.466 [0.419, 0.516] (R=1.000)* | 3.5 | 3.635 [3.267, 4.003] (R=1.000) | 0.9 | 0.867 [0.793, 0.945] (R=1.000) |
| 9 | 0.3 | 0.289 [0.230, 0.347] (R=1.000) | **0.7** | 0.640 [0.588, 0.688] (R=1.000)* | 3.5 | 3.501 [3.043, 3.914] (R=1.000) | 0.9 | 0.913 [0.839, 0.985] (R=1.001) |
| 10 | 0.3 | 0.269 [0.192, 0.345] (R=1.000) | 0.35 | 0.377 [0.313, 0.441] (R=1.000) | **2** | 2.061 [1.818, 2.283] (R=1.001) | 0.9 | 0.900 [0.838, 0.963] (R=1.000) |
| 11 | 0.3 | 0.255 [0.204, 0.306](R=1.000) | 0.35 | 0.343 [0.287, 0.394](R=1.000) | **2.75** | 2.904 [2.631, 3.144](R=1.001) | 0.9 | 0.931 [0.861, 1.003](R=1.000) |
| 12 | 0.3 | 0.287 [0.231, 0.342] (R=1.001) | 0.35 | 0.370 [0.329, 0.411]  R=1.000) | **4.25** | 4.325 [3.903, 4.705] (R=1.000) | 0.9 | 0.888 [0.798, 0.977] (R=1.000) |
| 13 | 0.3 | 0.286 [0.236, 0.335] (R=1.000) | 0.35 | 0.374 [0.335, 0.412] (R=1.000) | **5** | 4.975 [4.546, 5.406] (R=1.000) | 0.9 | 0.871 [0.774, 0.962] (R=1.000) |
| 14 | 0.3 | 0.307 [0.262, 0.351] (R=1.000) | 0.35 | 0.323 [0.285, 0.360] (R=1.000) | 3.5 | 3.554 [3.320, 3.766] (R=1.001) | **0.1** | 0.052 [-0.023, 0.127] (R=1.000) |
| 15 | 0.3 | 0.302 [0.251, 0.355] (R=1.000) | 0.35 | 0.365 [0.321, 0.408] (R=1.000) | 3.5 | 3.501 [3.218, 3.749] (R=1.001) | **0.5** | 0.476 [0.403. 0.553] (R=1.000) |
| 16 | 0.3 | 0.259 [0.199, 0.318] (R=1.000) | 0.35 | 0.367 [0.319, 0.415] (R=1.000) | 3.5 | 3.505 [3.137, 3.872] (R=1.000) | **1.3** | 1.351 [1.264, 1.433] (R=1.000) |
| 17 | 0.3 | 0.268 [0.200, 0.336] (R=1.000) | 0.35 | 0.345 [0.293, 0.397] (R=1.000) | 3.5 | 3.497 [3.096, 3.887] (R=1.000) | **1.7** | 1.661 [1.565, 1.757] (R=1.000) |

*There were no significant differences between real or simulated data for the accuracy in reward or punishment trials for both the placebo (reward trials: M_real_= 75.19(19.16), M_sim_=79.19(12.99), t(49.23)= -1.04, p=.31, d= .24; punishment trials: M_real_= 71.35(10.12), M_sim_=70.79(6.09), t(45.46)= .28, p= .78, d= .07) and the escitalopram groups (reward trials: M_real_= 79.17(18.31), M_sim_=75.63(15.24), t(80)= .95, p= .35, d= .22; punishment trials: M_real_=70.21(10.16), M_sim_=70.84(7.38), t(51.74)= -.31, p= .76, d= .07).*

**
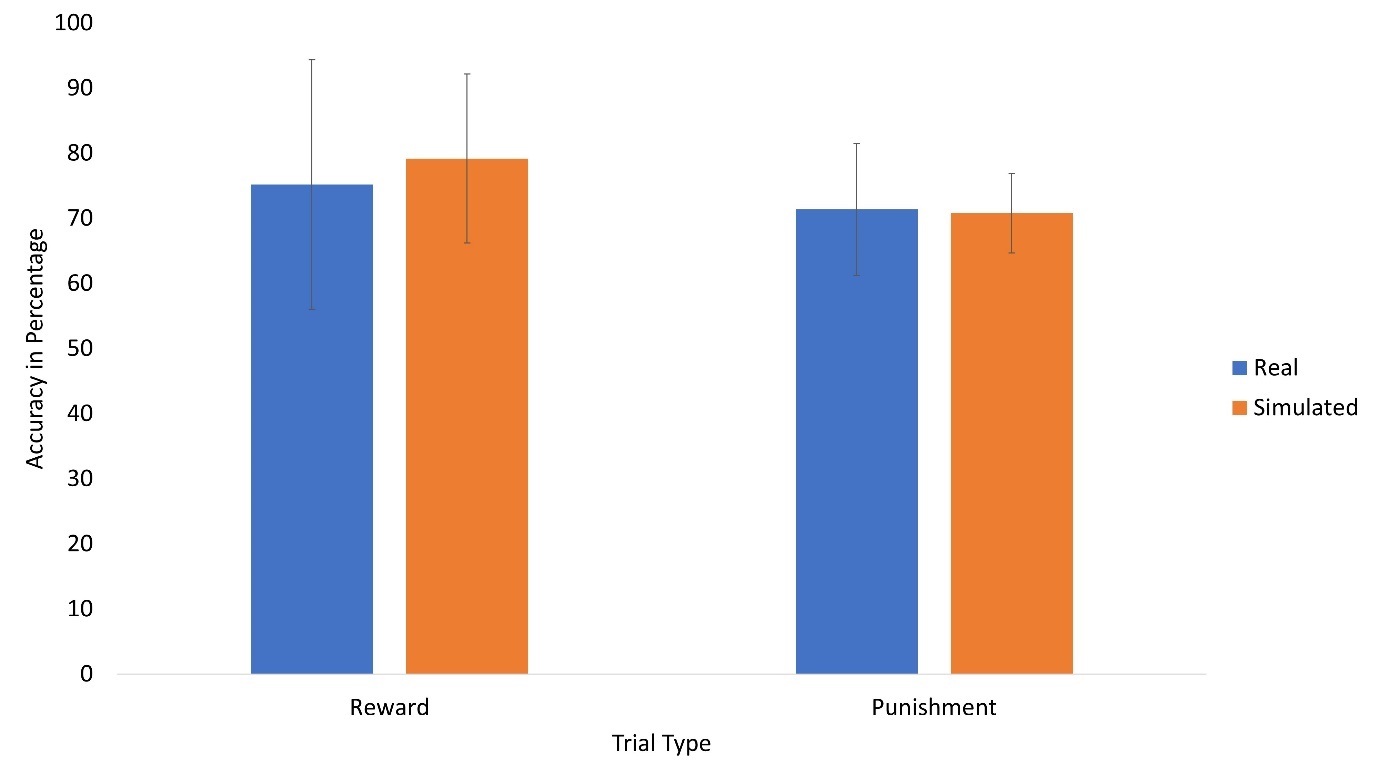
**

**Figure S4.** Real and simulated accuracy for the placebo group. *The real data is displayed in blue, and the simulated data is displayed in orange.*


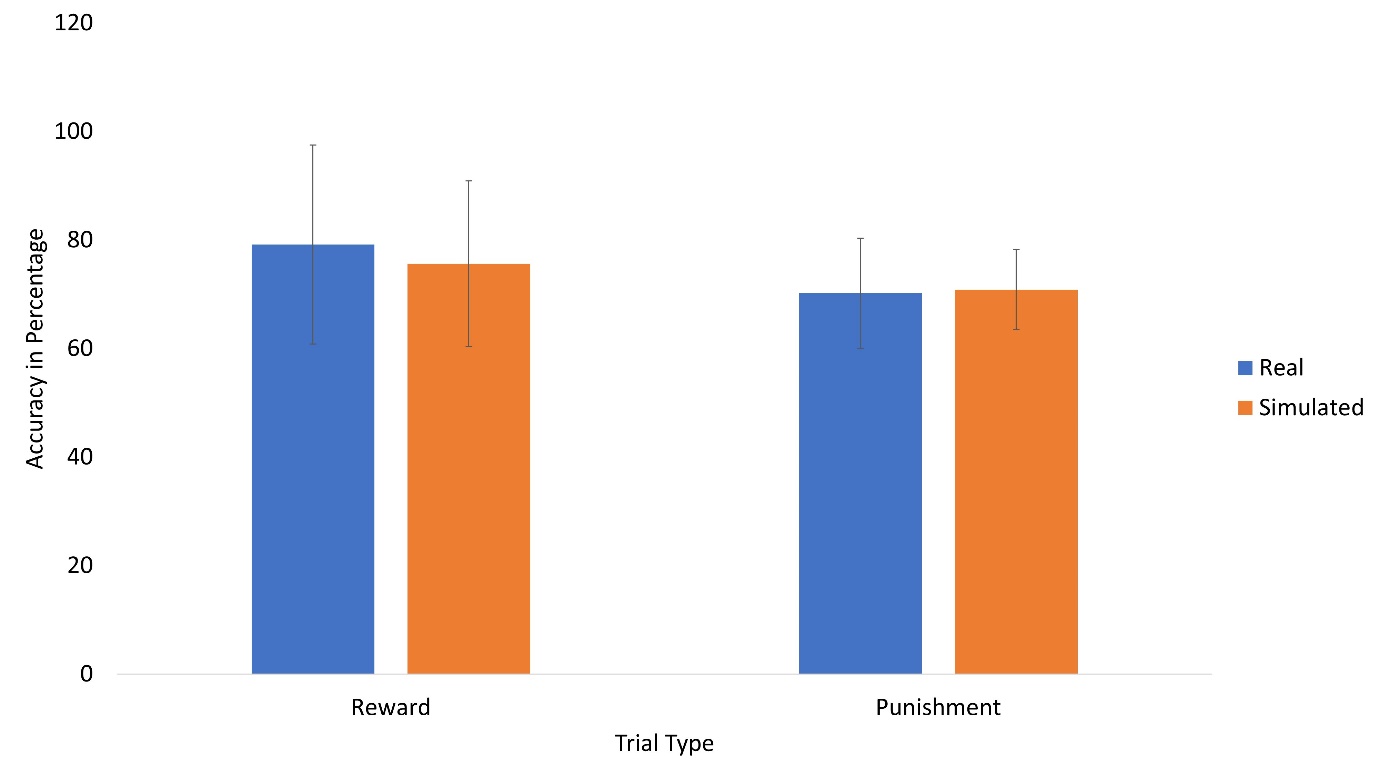


**Figure S5.** Real and simulated accuracy for the escitalopram group. *The real data is displayed in blue, and the simulated data is displayed in orange.*

### Neuroimaging results:

There were no significant differences in the neuronal activations between the placebo and escitalopram groups associated with either reward or punishment prediction errors.

# References

Christakou, A., Gershman, S. J., Niv, Y., Simmons, A., Brammer, M., & Rubia, K. (2013). Neural and psychological maturation of decision-making in adolescence and young adulthood. Journal of cognitive neuroscience, 25(11), 1807-1823.

Den Ouden, H. E., Daw, N. D., Fernandez, G., Elshout, J. A., Rijpkema, M., Hoogman, M., Franke, B., & Cools, R. (2013). Dissociable effects of dopamine and serotonin on reversal learning. *Neuron*, *80*(4), 1090-1100.

Gelman, A., Hill, J., & Yajima, M. (2012). Why we (usually) don't have to worry about multiple comparisons. *Journal of research on educational effectiveness*, *5*(2), 189-211.

Gershman, S. J. (2016). Empirical priors for reinforcement learning models. Journal of Mathematical Psychology, 71, 1-6.

Gronau, Q. F., Sarafoglou, A., Matzke, D., Ly, A., Boehm, U., Marsman, M., Leslie, D. S., Forster, J. J., Wagenmakers, E.-J., & Steingroever, H. (2017). A tutorial on bridge sampling. *Journal of mathematical psychology*, *81*, 80-97.

Kanen, J. W., Ersche, K. D., Fineberg, N. A., Robbins, T. W., & Cardinal, R. N. (2019). Computational modelling reveals contrasting effects on reinforcement learning and cognitive flexibility in stimulant use disorder and obsessive-compulsive disorder: remediating effects of dopaminergic D2/3 receptor agents. *Psychopharmacology*, *236*, 2337-2358.

Kruschke, J. K. (2010). Bayesian data analysis. *Wiley Interdisciplinary Reviews: Cognitive Science*, *1*, 658-676.

Langley, C., Armand, S., Luo, Q., Savulich, G., Segerberg, T., Søndergaard, A., Pedersen, E. B., Svart, N., Overgaard-Hansen, O., & Johansen, A. (2023). Chronic escitalopram in healthy volunteers has specific effects on reinforcement sensitivity: a double-blind, placebo-controlled semi-randomised study. *Neuropsychopharmacology*, *48*(4), 664-670.

Marzuki, A. A., Tomić, I., Ip, S. H. Y., Gottwald, J., Kanen, J. W., Kaser, M., Sule, A., Conway-Morris, A., Sahakian, B. J., & Robbins, T. W. (2021). Association of environmental uncertainty with altered decision-making and learning mechanisms in youths with obsessive-compulsive disorder. *JAMA network open*, *4*(11), e2136195-e2136195.
